# Temporal changes in the discrepancy between subjective and objective sleep: an in-home electroencephalography study

**DOI:** 10.3389/fpubh.2026.1863955

**Published:** 2026-07-07

**Authors:** Jaehoon Seol, Ryuji Ochiai, Atsushi Suzuki, Tadashi Hase, Kumpei Tokuyama, Toshio Kokubo, Tomohiro Okura, Masashi Yanagisawa

**Affiliations:** 1 College of Sport Science, Sungkyunkwan University, Suwon, Gyeonggi, Republic of Korea; 2 International Institute for Integrative Sleep Medicine (WPI-IIIS), Tsukuba Institute for Advanced Research (TIAR), University of Tsukuba, Tsukuba, Ibaraki, Japan; 3 R&D Center for Tailor-Made QOL, University of Tsukuba, Tsukuba, Ibaraki, Japan; 4 Department of Frailty Research, Center for Gerontology and Social Science, National Center for Geriatrics and Gerontology, Obu, Aichi, Japan; 5 Research and Development, Kao Corporation, Tokyo, Japan; 6 S’UIMIN, Inc., Tokyo, Japan; 7 R&D Center for Frontiers of Mirai in Policy and Technology (F-MIRAI), University of Tsukuba, Tsukuba, Ibaraki, Japan; 8 Institute of Health and Sport Sciences, University of Tsukuba, Tsukuba, Ibaraki, Japan; 9 Department of Molecular Genetics, University of Texas Southwestern Medical Center, Dallas, TX, United States

**Keywords:** delta power, electroencephalography, sleep diary, sleep misperception, sleep perception index, subjective sleep quality

## Abstract

**Background:**

Sleep misperception has traditionally been attributed to neurophysiological abnormalities or psychological traits; however, most evidence is based on single-night or laboratory-based assessments. This raises the possibility that misperception may partly reflect measurement limitations rather than stable individual pathology. This study aimed to examine whether sleep misperception changes across consecutive nights and to identify variability-based phenotypes using repeated in-home electroencephalography (EEG).

**Methods:**

Eighty-three adults (40–64 years) underwent 7 consecutive nights of in-home EEG monitoring and completed daily sleep questionnaires (OSA-MA). Sleep misperception was quantified using the Sleep Perception Index (SPI), and participants were classified as underestimators (*n* = 15), concordant estimators (*n* = 56), or overestimators (*n* = 12). Night-to-night variability in sleep parameters was assessed using coefficients of variation (CV).

**Results:**

Sleep misperception decreased over time, particularly among overestimators (*β* = −4.05, *p* < 0.001). Underestimators also showed an early correction by Night 2, although this change did not reach statistical significance. Compared with concordant estimators, overestimators exhibited greater variability in total sleep time (CV: 0.18 vs. 0.12, *p* = 0.033), whereas underestimators showed greater variability in sleep efficiency (CV: 0.11 vs. 0.06, *p* = 0.024) and N3 delta power (CV: 0.37 vs. 0.18, *p* = 0.003). Daily diary-based assessments (OSA-MA) demonstrated superior performance in identifying sleep-related discrepancies compared to retrospective measures.

**Discussion:**

These findings suggest that sleep misperception is not a fixed trait but a dynamic phenomenon that evolves with repeated measurement. Distinct variability patterns across sleep domains further indicate that misperception reflects instability in sleep quantity and quality rather than a single underlying mechanism. Multi-night, ethologically valid monitoring may therefore provide a more accurate framework for understanding and assessing sleep perception.

## 1 Introduction

Sleep is essential for physical, cognitive, and psychological health across all age groups (1–3). Insomnia—a common sleep disorder that affects approximately 30% of adults (with higher rates in older populations)—is associated with numerous adverse outcomes, including elevated cardiovascular risk, cognitive decline, and reduced quality of life (4–6). This disorder is typically evaluated using subjective instruments such as the Athens Insomnia Scale (AIS) (7). Sleep misperception—a persistent mismatch between individuals’ subjective sleep reports and objective sleep measures—has gained significant attention (8–10).

Sleep state misperception (also termed paradoxical insomnia) often manifests as a marked underestimation of sleep, wherein individuals report sleeping very little despite objectively normal or near-normal sleep architecture (8, 10). Conversely, some individuals perceive their sleep as adequate despite objectively short or fragmented sleep—representing an overestimation of sleep duration (8, 10). Thus, misperception represents a bidirectional mismatch between subjective and objective sleep (8). Polysomnography (PSG) and actigraphy studies have confirmed that individuals with insomnia tend to underestimate their total sleep time (TST) and overestimate sleep latency relative to objective recordings (11, 12). The mechanisms underlying this discrepancy remain unclear, although heightened nocturnal arousal and fragmentation of slow-wave (deep) sleep have been proposed as contributing factors (13–15).

To quantify the subjective–objective sleep gap, Lecci et al. (13) introduced the Sleep Perception Index (SPI), defined as the ratio of perceived to objectively measured TST. A value of 100% indicates perfect agreement between subjective and objective durations. This index provides a continuous nightly measure of perception accuracy and enables tracking of changes in misperception over time.

Despite the availability of such measures, most studies on sleep misperception have been limited by short monitoring periods (1–3 nights) in laboratory settings. Single-night studies are susceptible to artificial conditions and “first-night” effects that can distort typical sleep patterns and bias subjective perception (16–18). However, in a 30-day forced desynchrony study, a correlation was observed between objective and self-reported sleep latency, and individuals who perceived longer sleep durations exhibited greater slow-wave sleep and fewer awakenings (19). Nevertheless, the pattern of changes in subjective versus objective sleep misperception over consecutive nights in a natural environment remains unknown (16, 20, 21). Specifically, the physiological basis of persistent misperceptions remains unclear. Emerging evidence links higher slow-wave activity (delta power) with more accurate sleep perception, whereas fragmented deep sleep may contribute to subjective underestimation (22, 23). However, these possibilities have not been directly examined across multiple nights (24, 25).

Further, it remains unclear whether comprehensive assessments of subjective sleep quality can measure sleep discrepancies more effectively than traditional insomnia scales. Notably, AIS focuses mainly on sleep initiation and maintenance difficulties (26), potentially overlooking other dimensions of the sleep experience. Conversely, the Oguri–Shirakawa–Azumi Sleep Inventory for middle-aged individuals (OSA-MA) evaluates multiple facets of subjective sleep quality, including sleep onset/maintenance, dreams, refreshment upon awakening, and perceived sleep length, thereby providing a more comprehensive self-assessment (27, 28). Whether the results from such a multidimensional questionnaire align with insomnia classifications based on the AIS or correspond to objective sleep indices remains elusive. Notably, mismatches in perceived sleep quality have gained less attention than quantitative aspects, despite many individuals reporting feeling unrefreshed even with objectively sufficient sleep duration (29). This gap highlights the need to examine subjective–objective discrepancies in sleep quality as well as quantity.

To the best of our knowledge, this study is the first to examine the temporal dynamics of sleep misperception across multiple consecutive nights using in-home electroencephalography (EEG) in a home environment. In this exploratory study, we aimed to investigate these dynamics over seven consecutive nights using in-home EEG and daily sleep questionnaires. Specifically, we addressed three key questions: First, whether the magnitude of the subjective–objective sleep discrepancy diminishes over the course of 1 week of nightly monitoring; second, whether individuals who persistently underestimate their sleep exhibit greater night-to-night variability in deep sleep (slow-wave) parameters; and third, whether a multidimensional sleep quality questionnaire more effectively identifies poor sleepers than standard scales, such as the AIS and Epworth Sleepiness Scale (ESS).

## 2 Methods

### 2.1 Participants and study design

This micro-longitudinal observational study was conducted between December 2021 and September 2022. Inclusion criteria were: (1) age 40–64 years; (2) no current use of sleep-influencing medications, such as hypnotics, antipsychotics, anxiolytics, or antidepressants; and (3) habitual daily consumption of less than three cups of caffeinated beverages (such as coffee, green tea, or black tea).

A total of 86 participants were recruited via online advertisements and printed posters. After receiving a detailed explanation of the study procedure, participants provided written informed consent. Thereafter, they completed a baseline questionnaire assessing demographic information, insomnia symptoms (AIS), and daytime sleepiness (ESS). Subsequently, participants underwent 1 week of at-home sleep monitoring using a portable EEG device and completed a daily sleep questionnaire based on the OSA-MA. This duration was selected based on previous evidence suggesting that approximately 7 days are sufficient for reliable assessment of habitual sleep patterns (30).

This study was conducted in accordance with the principles of the Declaration of Helsinki and approved by the Ethics Review Committee of the Chiyoda Paramedical Care Clinic (Approval No. 21111903). The study was registered in the University Hospital Medical Information Network (UMIN000047139), and the data used in this analysis were derived from the baseline phase of a registered study.

### 2.2 Subjective sleep measures

Retrospective subjective sleep was assessed using the AIS and ESS. The AIS comprises eight items scored on a scale of 0–3 to evaluate sleep difficulties over the past 2 weeks. A total score of ≥6 indicates probable insomnia (7). The ESS assesses daytime sleepiness through eight items scored from 0–3, with a total score ≥11 suggesting excessive daytime sleepiness (31). Both scales have demonstrated reliability and validity in diverse populations (7, 32).

We assessed daily subjective sleep using the OSA-MA (28). This questionnaire captures self-reported sleep experiences upon awakening, including bedtime, waketime, sleep latency, nighttime awakenings, and perceived sleep duration. It comprises 16 items rated on a 4-point scale (0–3) grouped into five subscales: sleepiness on rising, sleep initiation and maintenance, frequent dreams, feeling refreshed, and sleep length. Each subscale score is standardized (mean = 50; higher scores indicate better subjective sleep), and a global score is calculated by summing across subscales.

### 2.3 Objective sleep measures

Objective sleep was measured using the Insomnograf K2 (S’UIMIN Inc., Tokyo, Japan)—a validated, lightweight (162 g) in-home EEG device with integrated frontal electrodes (10, 27, 33). The device records EEG signals via the Fp1, Fp2, M1, M2, and Fpz positions using a 10–20 system montage, enabling four derivations and additional channels for electrooculograms and submental electromyograms. Sleep stages (W, N1, N2, N3, and rapid eye movement [REM]) were scored manually in 30-s epochs by a certified sleep specialist according to the American Academy of Sleep Medicine criteria (34). Sleep latency, REM latency, TST, sleep efficiency, and wake after sleep onset (WASO) were derived accordingly.

For spectral analysis, delta power (0.5–4 Hz) was computed from preprocessed EEG signals (bandpass 0.3–45 Hz) using the Fast Fourier Transform and Welch’s method (27). Given the frontal electrode placement, delta activity was assessed primarily from the Fp1 and Fp2 derivations. Data processing was conducted using MNE-Python, SciPy, and related Python libraries.

### 2.4 Definition of sleep perception discrepancy

To examine sleep perception discrepancies, nightly SPI values were calculated as the ratio of subjective TST obtained from the OSA-MA questionnaire to objective TST derived from the corresponding night of in-home EEG monitoring. Higher SPI values indicate an overestimation of sleep duration, whereas lower values indicate an underestimation (13). Participants were classified into three perception groups based on the distribution of baseline (Day 1) SPI values: underestimators [SPI < mean − 0.5 standard deviation (SD); *n* = 15], concordant estimators (mean − 0.5 SD ≤ SPI < mean + 0.5 SD; *n* = 56), and overestimators (SPI ≥ mean + 0.5 SD; *n* = 12). Although this categorization was not derived from prior SPI-specific studies (13), similar SD-based criteria have been used to define perception accuracy in other misperception studies (35). Thus, this approach offers a pragmatic method for sleep perception classification, enabling the detection of meaningful intergroup differences while maintaining sufficient statistical power.

### 2.5 Covariates

Potential confounding variables included continuous measures [age and body mass index (BMI)] and categorical factors. Binary variables were assessed via self-reports and included sex (male/female), smoking status (current vs. never/previous), alcohol consumption (yes/no), caffeine intake (yes/no), and medical history (presence/absence of sleep disorders, hypertension, diabetes, dyslipidemia, and hyperuricemia). For each item, participants indicated whether they had received a prior diagnosis or treatment.

### 2.6 Statistical analysis

To examine temporal changes in SPI over the 7-day period, a linear mixed-effects model was used, with day, perception group, and their interaction as fixed effects and participant ID as a random intercept. Raw SPI values were entered into the model without centering, transformation, or scaling. Adjusted daily means and 95% confidence intervals (CIs) of SPI were computed for each group.

To evaluate the variability of objective sleep parameters, the coefficient of variation (CV) for each EEG-derived sleep parameter (including TST and sleep stages such as N1, N2, N3, and REM) was calculated across the 7 days for each participant. One-way analysis of variance (ANOVA) was subsequently conducted to compare CVs across the three SPI-based groups, followed by Bonferroni-adjusted post-hoc comparisons.

Participants were further classified based on AIS (≥6 vs. <6) and ESS (≥11 vs. <11) scores (7, 32). Sleep parameters measured using the EEG device were compared between these groups using independent sample t-tests. To assess the validity of the OSA-MA in detecting subjective sleep complaints, we conducted receiver operating characteristic (ROC) analyses using AIS and ESS thresholds as reference standards. Logistic regression models were fitted for each OSA-MA subscale and the total score after adjusting for sex, age, BMI, alcohol consumption, smoking status, and caffeine intake. From these models, propensity scores, area under the curve (AUC), sensitivity, specificity, and cutoff values were derived. The agreement between OSA-MA-based classifications and AIS/ESS-defined insomnia or excessive daytime sleepiness was assessed using Cohen’s kappa coefficients. Finally, participants were categorized as good or poor sleepers based on the newly derived OSA-MA total score cutoff, and EEG-derived sleep parameters were compared between these groups using t-tests.

All statistical analyses were performed using Python (version 3.12.2) and Visual Studio Code (version 1.87.2). A two-tailed *p*-value < 0.05 was considered significant.

## 3 Results

Of the 86 participants who provided consent, three were excluded from the analysis because they had <4 days of in-home EEG device use and daily sleep assessments. Table 1 presents the demographic and clinical characteristics of the study participants (*n* = 83). The mean age was 52.2 years (SD = 6.7), while the average BMI was 23.0 kg/m^2^ (SD = 3.1). Among the participants, 47.0% were female, 21.7% reported alcohol consumption, and 4.8% were current smokers. Based on the AIS, 65.1% of participants scored ≥6, indicating probable insomnia, while 45.8% scored ≥11 on the ESS, suggesting excessive daytime sleepiness. The average number of valid in-home EEG recording nights was 6.6 out of 7. The mean TST measured via in-home EEG was 345.8 min, with a sleep efficiency of 87.8%. The mean percentage of N3 sleep was 10.2%, and the total delta power during N3 averaged 478.8 μV^2^.

**Table 1:** Participant (*n* = 83) characteristics.

Parameter	Mean	±	SD	Min	Max
Age (years)	52.2 ± 6.7			40	64
BMI, kg/m^2^	23.0 ± 3.1			16.8	34.4
Female, *n* (%)	39	(47.0)			
Alcohol consumption (drinker), *n* (%)	18	(21.7)			
Tobacco-smoking status (smoker), *n* (%)	4	(4.8)			
AIS, score	7.8 ± 4.8			0	17.0
Insomnia (≥6), *n* (%)	54	(65.1)			
ESS, score	9.6 ± 6.1			0	22.0
Excessive sleepiness (≥11), *n* (%)	38	(45.8)			
No. of days with valid in-home EEG and OSA-MA, (days)	6.6 ± 0.8			4	7
In-home EEG device sleep parameters averaged over 1 week					
Total bedtime, min	374.1 ± 56.7			221.9	484.4
TST, min	345.8 ± 50.5			246.8	464.5
Sleep latency, min	19.0 ± 17.8			1.9	114.4
Sleep efficiency, %	87.8 ± 6.6			59.7	96.5
REM latency, min	67.1 ± 21.6			23.9	119.4
Arousal index	12.8 ± 4.9			5.1	25.8
N1, %	8.9 ± 4.0			1.7	20.8
N2, %	49.2 ± 6.1			36.6	62.9
N3, %	10.2 ± 7.3			0.0	27.2
REM, %	24.1 ± 4.0			15.7	35.4
WASO, %	7.6 ± 4.6			1.7	21.7
Total delta power during N1, μV^2^	65.3 ± 31.5			23.8	217.1
Total delta power during N2, μV^2^	106.9 ± 40.8			44.5	227.8
Total delta power during N3, μV^2^	478.8 ± 301.2			181.1	2335.1
Total delta power during REM, μV^2^	44.8 ± 16.8			21.6	121.8
OSA-MA scores averaged over 1 week					
Sleepiness on rising	44.3 ± 9.1			22.0	65.0
Initiation and maintenance of sleep	41.3 ± 8.1			22.2	60.7
Frequent dreaming	48.6 ± 7.9			28.7	58.4
Refreshing	44.8 ± 9.1			22.6	63.7
Sleep length	43.5 ± 8.9			21.5	64.6
Global OSA-MA score	222.5 ± 34.2			130.1	286.0

SD, standard deviation; EEG, electroencephalography; OSA-MA, Oguri–Shirakawa–Azumi sleep inventory, Middle-Aged and Aged version; REM, rapid-eye-movement sleep; WASO, wake after sleep onset; TST, total sleep time; AIS, Athens insomnia scale; Epworth sleepiness scale; N1, night 1; N2, night 2; N3, night 3; n, number.

**Figure 1:**
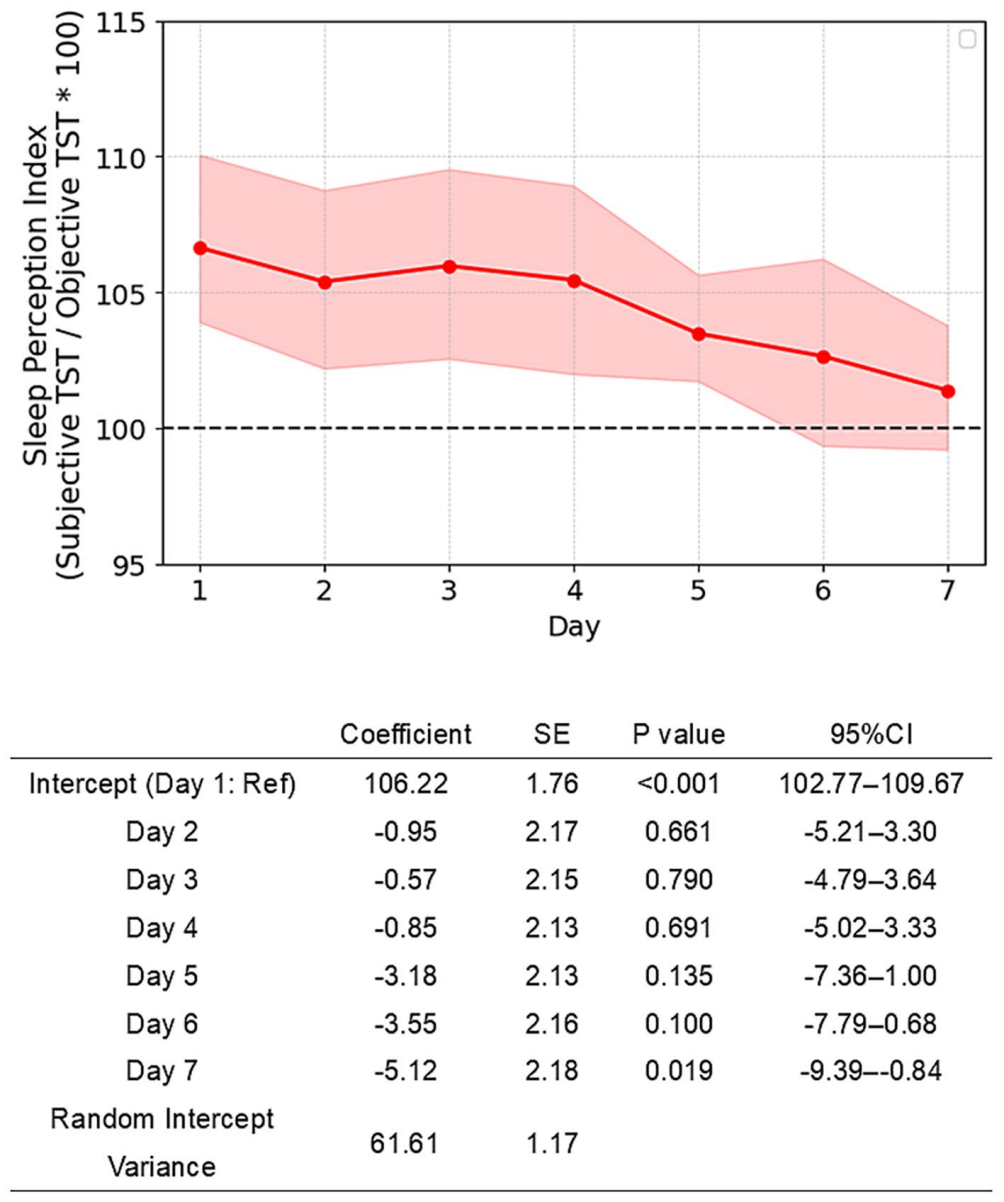
Changes in the Sleep Perception Index over seven consecutive nights. The red line represents the adjusted mean Sleep Perception Index (subjective TST/objective TST × 100) with 95% CI (shaded area) from a linear mixed-effects model with random intercepts. The dashed line indicates perfect agreement (Sleep Perception Index = 100%). TST, total sleep time; CI, confidence interval.

Figure 1 illustrates day-to-day changes in SPI across seven consecutive nights. A significant downward trend was observed, indicating improved alignment between subjective and objective TST. Linear mixed-effects modeling revealed a significant reduction in SPI by Day 7 (*β* = −5.12; *p* = 0.019), relative to Day 1 (Figure 1).

**Figure 2:**
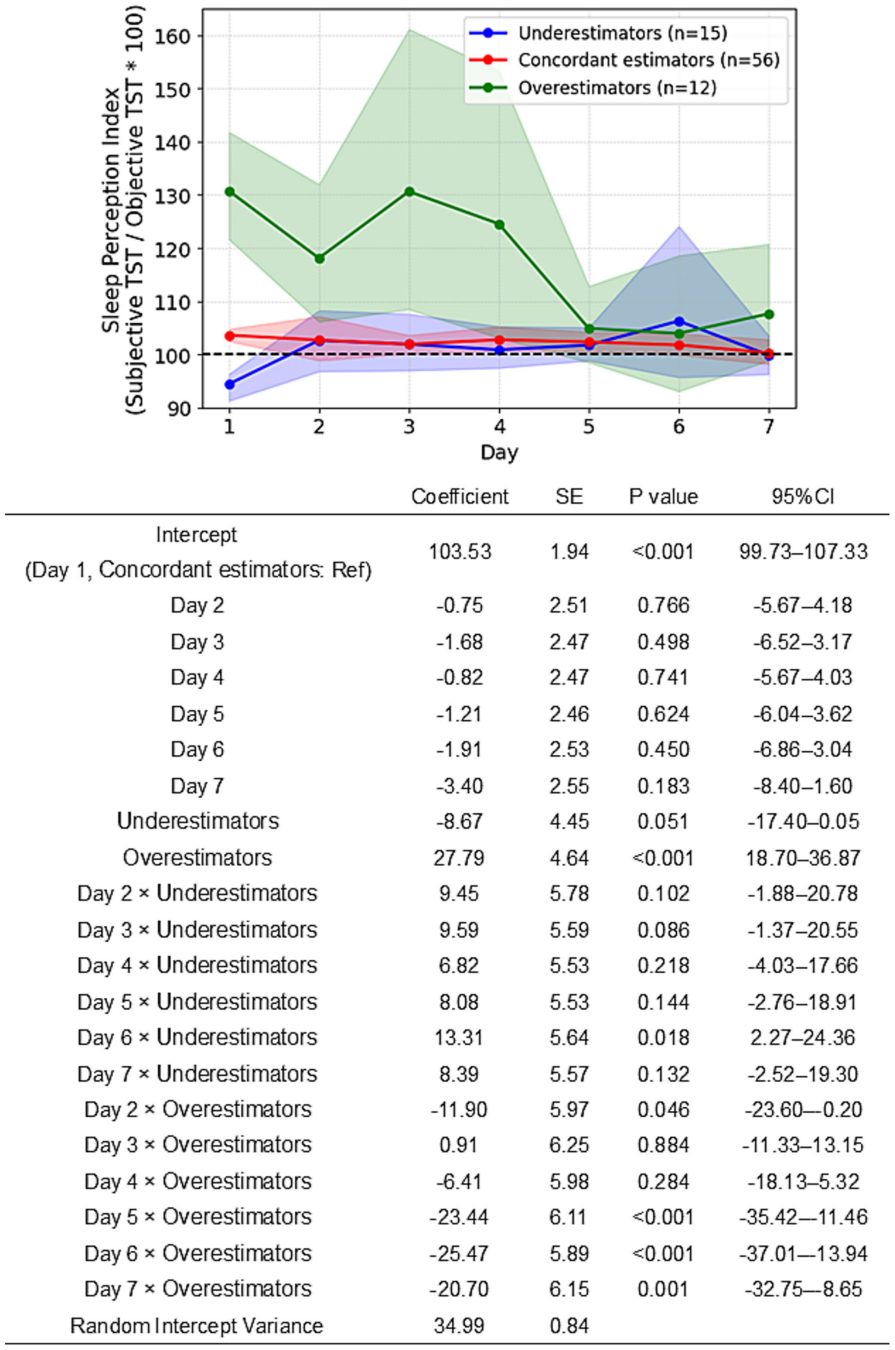
Changes in the sleep perception index over seven consecutive nights stratified by baseline perception groups. Colored lines represent the adjusted mean sleep perception index (subjective TST/objective TST × 100) with a 95% CI (shaded areas) from a linear mixed-effects model with random intercepts. Group classification was based on the baseline Sleep Perception Index as follows: underestimators (*n* = 15), concordant estimators (*n* = 56), and overestimators (*n* = 12). The dashed line indicates perfect agreement (SPI = 100%). TST, total sleep time; CI, confidence interval.

Figure 2 displays the SPI trajectories across the three groups, classified by initial SPI values. Overestimators demonstrated a pronounced decline in SPI over time, suggesting a progressive correction in perception, whereas underestimators and concordant estimators showed relatively stable SPI trajectories throughout the 7-day period.

Furthermore, Table 2 presents the mixed-effects model results for SPI. Overestimators showed significantly higher SPI values at baseline than concordant estimators (*β* = 31.27, *p* < 0.001). A significant interaction was observed for overestimators, indicating a greater decline in SPI over time compared with concordant estimators (Time × Overestimators: *β* = −4.05, *p* < 0.001). No significant interaction effect was observed for underestimators (Time × Underestimators: *β* = 1.04, *p* = 0.163).

**Table 2:** Group differences and time trends in sleep perception index across seven consecutive nights.

Variables	Coefficient	SE	*p*-value	95% CI
Intercept (Day 1, concordant estimators: Ref)	103.84	1.71	<0.001	100.49–107.19
Underestimators (vs. concordant estimators)	−4.94	3.84	0.199	−12.48–2.60
Over estimators (vs. concordant estimators)	31.27	4.14	<0.001	23.16–39.38
Time (per day, concordant estimators)	−0.42	0.34	0.215	−1.09–0.25
Time × under estimators (vs. concordant estimators)	1.04	0.75	0.163	−0.42–2.51
Time × over estimators (vs. concordant estimators)	−4.05	0.82	<0.001	−3.66–-2.44

Estimates were derived from a linear mixed-effects model with random intercepts. The intercept represents the estimated SPI value for concordant estimators on Day 1 (reference group). “Time” represents the daily change in SPI among concordant estimators, and interaction terms represent differences in daily change relative to concordant estimators. Group classification was based on baseline (Day 1) Sleep Perception Index values. SE, standard error; CI, confidence interval.

**Figure 3:**
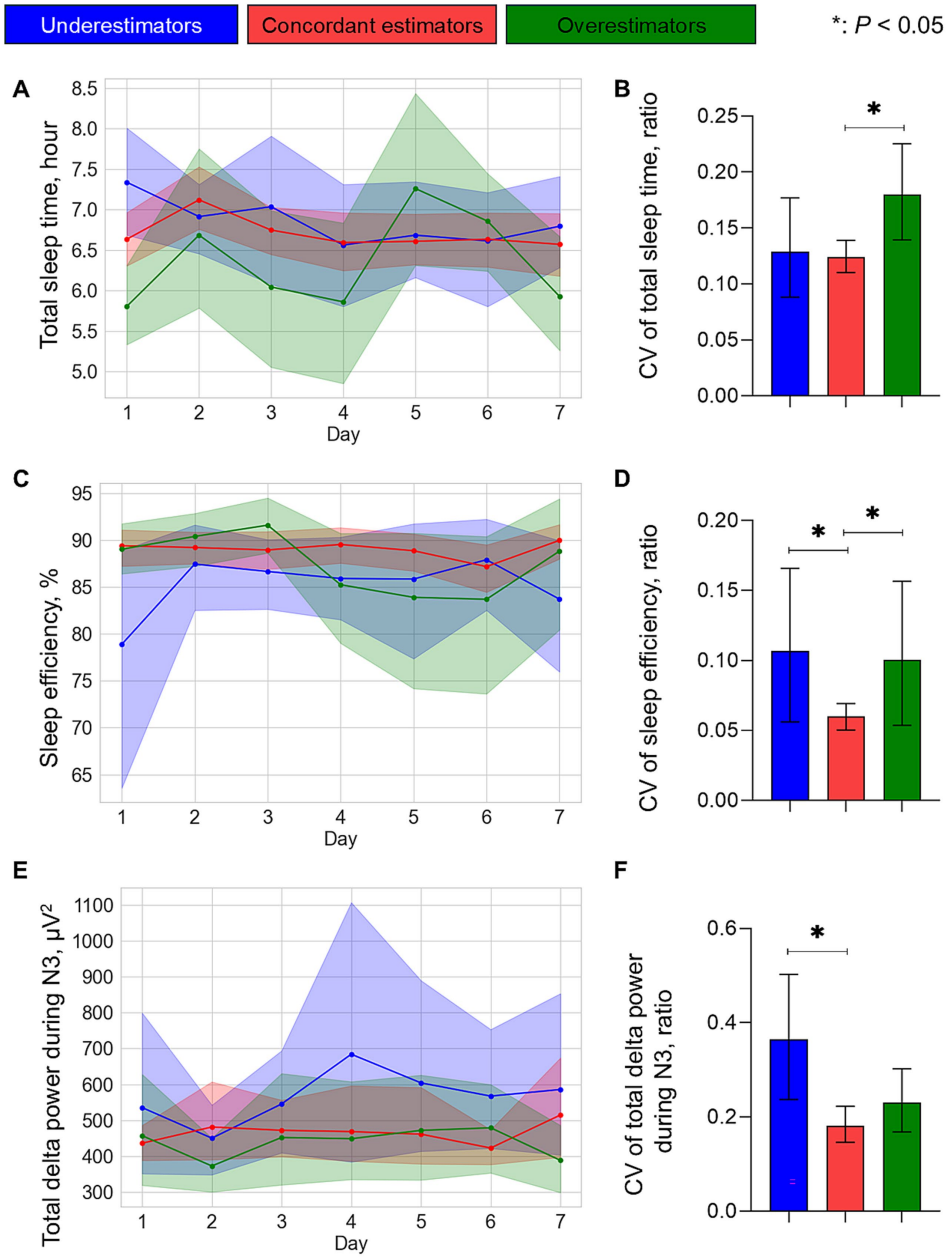
Night-to-night variability in TST, sleep efficiency, and total delta power during N3 sleep stratified by baseline perception groups. Panels **(A)**, **(C)**, and **(E)** show the adjusted mean values (±95% CI) for EEG-derived objective TST (hours), sleep efficiency (%), and total N3 delta power (μV^2^), respectively, over 7 nights. Panels **(B)**, (**D**), and **(F)** show the coefficient of variation (CV) of the corresponding EEG-derived parameters across the 7 nights. Group classification was based on the baseline SPI: underestimators (blue), concordant estimators (red), and overestimators (green). *p < 0.05, Bonferroni-corrected *post hoc* test following one-way ANOVA. Results for the other sleep parameters are presented in Supplementary Table S1. TST, total sleep time; CV, coefficient of variation; CI, confidence interval; ANOVA, analysis of variance.

Only sleep parameters showing significant between-group differences in variability were included in Figure 3; non-significant results are provided in Supplementary Table S1. Figure 3A illustrates the day-to-day pattern of TST across the 7 days, whereas Figure 3B shows the corresponding variability quantified by CV. Significant group differences in variability were observed (ANOVA *p* = 0.033) (Figure 3B). Specifically, the CV of TST was significantly higher in overestimators than in concordant estimators (*p* = 0.005). Figure 3C displays the day-to-day pattern of sleep efficiency, while Figure 3D presents the between-group differences in variability (ANOVA *p* = 0.024). The CV of sleep efficiency was significantly higher in both underestimators (*p* = 0.011) and overestimators (*p* = 0.017) compared with concordant estimators. Figure 3E illustrates the night-to-night pattern of total delta power, and Figure 3F shows the corresponding variability across groups (ANOVA *p* = 0.003). The CV was significantly higher in underestimators than in concordant estimators (*p* = 0.001).

Table 3 compares the “first-night” EEG sleep data between groups categorized according to AIS and ESS scores. Participants with probable insomnia (AIS ≥ 6) showed significantly lower sleep efficiency (86.6% vs. 90.0%; *p* = 0.023) and higher WASO (8.5% vs. 6.1%; *p* = 0.026) than those without insomnia. No significant group differences were observed for ESS-based classification in EEG parameters, although excessive sleepiness (ESS ≥ 11) showed a trend toward higher WASO (8.5% vs. 6.9%; *p* = 0.098).

**Table 3:** Comparison of first-night in-home EEG sleep data between groups based on Athens insomnia scale and Epworth sleepiness scale criteria.

Sleep parameter	Athens insomnia scale			Epworth sleepiness scale		
	Non-insomnia group (<6)	Insomnia group (≥6)	Unpaired *t* test *p*-value	Normal sleepiness group (<11)	Excessive sleepiness group (≥11)	Unpaired *t test* *p*-value
	*n* = 29	*n* = 54		*n* = 45	*n* = 38	
	Mean ± SD	Mean ± SD		Mean ± SD	Mean ± SD	
Total bedtime, min	378.5 ± 47.8	371.8 ± 61.2	0.610	377.5 ± 53.4	370.2 ± 60.8	0.561
Total sleep time, min	356.0 ± 47.8	340.3 ± 51.5	0.179	351.3 ± 49.6	339.2 ± 51.5	0.277
Sleep latency, min	15.3 ± 22.0	20.9 ± 14.9	0.169	17.5 ± 19.2	20.7 ± 16.1	0.416
Sleep efficiency, %	90.0 ± 7.3	86.6 ± 5.9	0.023	88.6 ± 7.3	86.9 ± 5.7	0.255
REM latency, min	69.8 ± 24.1	65.7 ± 20.2	0.411	67.7 ± 23.9	66.3 ± 18.8	0.771
Arousal index, index	12.2 ± 4.0	13.1 ± 5.3	0.423	12.3 ± 4.3	13.3 ± 5.5	0.377
N1, %	8.6 ± 3.1	9.0 ± 4.4	0.584	8.4 ± 3.9	9.5 ± 4.1	0.222
N2, %	50.4 ± 4.7	48.6 ± 6.6	0.197	50.0 ± 5.7	48.2 ± 6.4	0.186
N3, %	10.3 ± 6.2	10.1 ± 7.9	0.902	10.1 ± 6.9	10.2 ± 7.9	0.955
REM, %	24.7 ± 3.8	23.9 ± 4.0	0.381	24.6 ± 3.9	23.6 ± 4.0	0.254
WASO, %	6.1 ± 3.7	8.5 ± 4.9	0.026	6.9 ± 4.4	8.5 ± 4.8	0.119
Total delta power during N1, μV^2^	62.2 ± 30.2	66.9 ± 32.3	0.525	65.6 ± 36.4	64.8 ± 25.0	0.908
Total delta power during N2, μV^2^	101.5 ± 38.2	109.8 ± 42.2	0.380	102.9 ± 41.2	111.5 ± 40.4	0.342
Total delta power during N3, μV^2^	458.4 ± 254.3	490.4 ± 326.9	0.657	475.8 ± 360.8	482.0 ± 224.6	0.928
Total delta power during REM, μV^2^	42.8 ± 13.4	45.9 ± 8.5	0.430	43.3 ± 15.4	46.5 ± 18.5	0.404

SD, standard deviation; REM, rapid-eye-movement sleep; N, non-REM sleep; WASO, wake after sleep onset.

**Figure 4:**
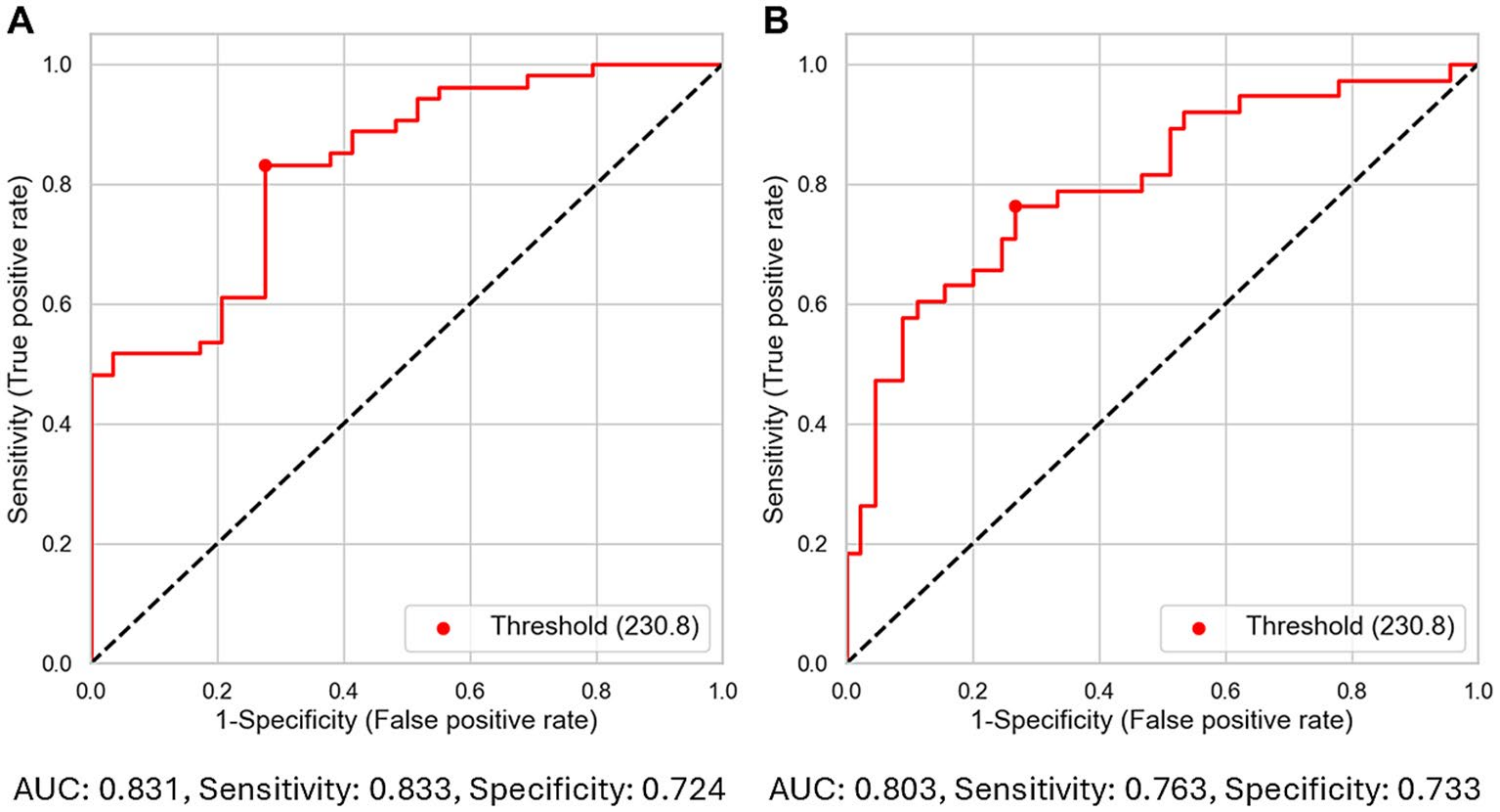
ROC curves of the OSA-MA total score for detecting subjective sleep complaints. Panel **(A)** shows the ROC curve for detecting probable insomnia defined by the AIS (≥6). Panel **(B)** shows the ROC curve for detecting excessive daytime sleepiness defined by the ESS (≥11). The red dot indicates the optimal cut-off point for the OSA-MA total score (230.8). ROC, receiver operating characteristic; AUC, area under the curve; AIS, Athens Insomnia Scale; ESS, Epworth Sleepiness Scale; OSA-MA, Oguri–Shirakawa–Azumi sleep inventory for Middle-aged Individuals. The value of 230.8 represents the optimal OSA-MA total score cutoff derived from ROC analysis and should not be interpreted as the AIS or ESS threshold.

Figure 4 shows the ROC curves using the AIS (Panel A) and ESS (Panel B) as reference criteria. The global OSA-MA score demonstrated strong predictive accuracy for both insomnia (AUC = 0.831, sensitivity = 0.833, specificity = 0.724) and excessive sleepiness (AUC = 0.803, sensitivity = 0.763, specificity = 0.733). For both models, ROC analysis identified an optimal OSA-MA total score cutoff of 230.8 for discriminating participants classified using the predefined AIS (≥6) and ESS (≥11) thresholds. The agreement between OSA-MA-based classification and AIS/ESS-defined clinical categories was moderate, with Cohen’s kappa coefficients of 0.486 (*p* < 0.001) for the AIS and 0.384 (*p* < 0.001) for the ESS.

To further examine the clinical utility of the OSA-MA cutoff score, participants were classified as good sleepers (OSA-MA total score ≥230.8) or poor sleepers (<230.8). As presented in Table 4, poor sleepers exhibited significantly longer sleep latency (23.0 vs. 12.8 min, *p* = 0.003), lower sleep efficiency (86.2% vs. 90.2%, *p* = 0.004), higher the arousal index (13.7 vs. 11.3, *p* = 0.019), and greater WASO (8.5% vs. 6.4%, *p* = 0.049).

**Table 4:** Comparison of 1-week averaged in-home EEG data based on novel cutoff values of the global OSA-MA score derived from the Athens insomnia scale and Epworth sleepiness scale.

	OSA-MA		
	Good sleeper (≥230.8)	Poor sleeper (<230.8)	Unpaired *t* test *p*-value
Sleep parameter	*n* = 33	*n* = 50	
	Mean ± SD	Mean ± SD	
Total bedtime, min	369.0 ± 57.5	377.5 ± 56.5	0.508
TST, min	346.1 ± 48.1	345.5 ± 52.5	0.958
Sleep latency, min	12.8 ± 10.0	23.0 ± 20.6	0.003
Sleep efficiency, %	90.2 ± 5.6	86.2 ± 6.8	0.006
REM latency, min	65.4 ± 24.7	68.2 ± 19.5	0.567
Arousal index, index	11.3 ± 3.7	13.7 ± 5.3	0.019
N1, %	7.7 ± 2.6	9.7 ± 4.6	0.015
N2, %	50.4 ± 6.4	48.4 ± 5.7	0.138
N3, %	10.0 ± 6.8	10.3 ± 7.7	0.872
REM, %	25.5 ± 3.3	23.2 ± 4.1	0.010
WASO, %	6.4 ± 4.1	8.5 ± 4.8	0.049
Total delta power during N1, μV^2^	61.7 ± 19.6	67.6 ± 37.4	0.357
Total delta power during N2, μV^2^	103.5 ± 34.8	109.1 ± 44.5	0.524
Total delta power during N3, μV^2^	483.2 ± 391.2	475.8 ± 226.2	0.917
Total delta power during REM, μV^2^	44.6 ± 15.9	44.9 ± 17.6	0.950

SD, standard deviation; OSA-MA, Oguri–Shirakawa–Azumi sleep inventory, Middle-Aged and Aged version; REM, rapid-eye-movement sleep; N, non-REM sleep; WASO, wake after sleep onset; TST, total sleep time.

## 4 Discussion

This exploratory study investigated intra-individual changes in subjective–objective sleep discrepancy across 7 nights of in-home EEG monitoring and examined associated variability in sleep patterns and continuity. By focusing on the temporal dimension, we aimed to clarify whether sleep misperception changes over days and to identify potential physiological signatures of persistent misperception. The findings revealed three key insights.

Notably, although participants were not clinically diagnosed with sleep disorders, they exhibited heterogeneous sleep characteristics, with a high prevalence of insomnia and daytime sleepiness. This heterogeneity may enhance the ecological validity of the findings by reflecting real-world variations in sleep perception. First, overestimators of sleep duration demonstrated a significant reduction in SPI across the week, suggesting that repeated objective measurements may facilitate the recalibration of self-assessments of sleep (13, 20). This finding suggests that baseline perception type is crucial in determining how subjective–objective alignment evolves over time (13, 36). Potential psychological mechanisms include anchoring bias, regression to the mean, and familiarity effect with monitoring devices or diary-based evaluations (37–39). Notably, improvement occurred even without structured behavioral intervention, implying that repeated self-monitoring may exert mild therapeutic effects on perceptual accuracy (40, 41). These results are consistent with prior studies demonstrating that longitudinal assessments can mitigate “first-night” effects and better capture habitual sleep patterns (20, 21).

Second, underestimators exhibited greater night-to-night variability in both sleep efficiency and total delta power during N3 sleep than concordant estimators. Such variability may contribute to the persistent underestimation of sleep, possibly because of inconsistent sleep quality or depth (42). Although previous studies have linked sleep misperception to elevated high-frequency EEG activity (including beta and gamma bands) (13, 14, 36, 43), the portable EEG system used in this study was optimized for sleep staging and provides more reliable measurements of slow-wave activity than of higher-frequency EEG activity. Therefore, analyses were restricted to delta-band activity. Consequently, the relative contribution of low-frequency and high-frequency EEG activity to sleep misperception could not be determined in the present study. Nevertheless, greater variability in N3 delta power among underestimators suggests that instability in sleep depth may be one neurophysiological feature associated with persistent sleep misperception (13, 44). Although underestimators showed a tendency toward early improvement, the discrepancy remained relatively stable throughout the monitoring period, suggesting a more entrenched mismatch between subjective and objective sleep that may require targeted interventions.

Third, our findings emphasize the clinical utility of diary-based assessments such as the OSA-MA questionnaire in capturing night-to-night fluctuations in misperception. Compared with retrospective tools such as the AIS and ESS, OSA-MA-based groupings—supported by newly established cutoff values calculated through ROC analyses—revealed more robust differences in EEG-derived features, including sleep efficiency and arousal index (7, 28), supporting previous recommendations for dynamic daily evaluations as a more sensitive approach for identifying maladaptive perception patterns and misperception phenotypes (45).

Notably, night-to-night variability (CV) in sleep parameters may serve as a physiological marker for differentiating misperception subtypes. Bonferroni-corrected post-hoc tests indicated that over estimators showed significantly higher CV in TST, whereas both over estimators and under estimators displayed elevated CV in sleep efficiency, and under estimators exhibited greater CV in delta power during N3 sleep than concordant estimators. These findings suggest that CV in different sleep domains characterizes distinct misperception phenotypes: overestimation of unstable sleep quantity (TST) and underestimation of unstable sleep quality and depth (efficiency and delta power) (46). Such patterns may reflect underlying instability in sleep patterns influenced by cognitive-emotional factors (hyperarousal) or unmeasured comorbidities (46). Moreover, CV metrics may serve not only as correlates but also as candidate biomarkers for identifying individuals prone to maladaptive sleep evaluation (47).

This study had some limitations. First, although we used the AIS because of its alignment with the International Classification of Sleep Disorders, third edition diagnostic criteria, alternative instruments such as the Insomnia Severity Index and Pittsburgh Sleep Quality Index may offer complementary insights (7, 48, 49). Second, conducting the study in a home-based setting limited control over external influences, although the daily questionnaires appeared to mitigate some overestimation effects. Third, the assessment period was relatively short; however, longer-term monitoring might yield different insights. Extended studies have shown stronger agreement among actigraphy, PSG, and sleep diaries (19, 30, 50). Fourth, we employed the OSA-MA, a validated tool developed in Japan that allows multidimensional assessment of sleep quality and quantity. Although this is a notable strength, its use may limit generalizability; validation against internationally recognized instruments such as the Consensus Sleep Diary (45) is warranted. Finally, the relatively small sample size of the overestimator subgroup (*n* = 12) may have reduced statistical stability and limited the generalizability of subgroup-specific findings. In addition, the micro-longitudinal design with a relatively short observation period warrants replication in larger cohorts with extended longitudinal follow-up and test–retest analyses.

In summary, this exploratory study highlights that misperception trajectories differ by baseline type; overestimators show perceptual recalibration with repeated monitoring, whereas underestimators maintain persistent discrepancies accompanied by physiological variability. Together with the superiority of daily diary-based tools, these findings underscore the value of longitudinal, ecologically valid monitoring for identifying distinct misperception phenotypes, ultimately providing a foundational basis for more personalized approaches to assessment and intervention.

## 5 Conclusion

This exploratory study showed that subjective–objective sleep discrepancy changes over consecutive nights in a naturalistic setting. Overestimators improved alignment with repeated monitoring, whereas underestimators maintained discrepancies along with greater variability in efficiency and N3 delta power. Daily multidimensional assessments (OSA-MA) identified perception discrepancies better than retrospective tools. These findings suggest that sleep misperception may not represent a fixed trait, but rather a dynamic phenomenon influenced by repeated measurement and individual variability in sleep quantity and quality. Multi-night, in-home EEG monitoring may therefore provide a more informative framework for identifying distinct sleep misperception phenotypes and developing personalized assessment and intervention strategies.

## Data Availability

The data used in this study were approved by Kao Corporation. The data are not publicly available due to proprietary restrictions but may be used for the future development of medical devices and diagnostic technologies. Requests regarding data access should be directed to the corresponding author.
