# Supplementary material for: Temporal changes in the discrepancy between subjective and objective sleep: an in-home electroencephalography study
Source: Front Public Health. 2026 Jul 7;14:1863955. doi: 10.3389/fpubh.2026.1863955 (PMC13386332; doi:10.3389/fpubh.2026.1863955)
Supplement: Supplementary file 1 [file Table_1.DOCX]

**Supplementary Information**

**Temporal changes in the discrepancy between subjective and objective sleep: An in-home electroencephalography study**

Jaehoon Seol, Ryuji Ochiai, Atsushi Suzuki, Tadashi Hase, Kumpei Tokuyama, Toshio Kokubo, Tomohiro Okura, and Masashi Yanagisawa

Correspondence should be addressed to Masashi Yanagisawa

Email: [yanagisawa.masa.fu@u.tsukuba.ac.jp](mailto:yanagisawa.masa.fu@u.tsukuba.ac.jp)

This PDF file includes:

Table S1

**Supplementary Table S1.** Day-by-day means, 95% confidence intervals, and coefficients of variation (CVs) for sleep parameters across perception groups over 7 nights

|  |  | Mean (95%CI) | | | | | | | CV mean (95%CI) | CV ANOVA P Value |
| --- | --- | --- | --- | --- | --- | --- | --- | --- | --- | --- |
|  | Group | Day 1 | Day 2 | Day 3 | Day 4 | Day 5 | Day 6 | Day 7 |  |  |
| Total bedtime, hour | Underestimators | 8.51  (7.70, 9.30) | 7.74  (7.16, 8.36) | 8.20  (7.06, 9.27) | 7.49  (6.72, 8.27) | 7.67  (6.90, 8.61) | 7.51  (6.49, 8.30) | 7.86  (6.96, 8.73) | 0.13  (0.09, 0.18) | 0.127 |
|  | Concordant estimators | 7.30  (6.91, 7.63) | 7.81  (7.44, 8.23) | 7.51  (7.21, 7.87) | 7.27  (6.81, 7.67) | 7.49  (7.06, 7.91) | 7.45  (6.98, 7.86) | 7.26  (6.73, 7.74) | 0.12  (0.11, 0.14) |  |
|  | Overestimators | 6.70  (6.12, 7.35) | 7.34  (6.41, 8.36) | 6.72  (5.57, 7.64) | 6.65  (5.68, 7.83) | 8.04  (7.00, 9.24) | 7.91  (7.11, 8.79) | 6.47  (5.61, 7.38) | 0.16  (0.12, 0.21) |  |
| Total sleep time, hour | Underestimators | 7.34  (6.72, 7.97) | 6.91  (6.47, 7.37) | 7.04  (6.19, 7.99) | 6.56  (5.82, 7.28) | 6.68  (6.09, 7.26) | 6.61  (5.84, 7.21) | 6.79  (6.23, 7.35) | 0.13  (0.09, 0.18) | 0.033 |
|  | Concordant estimators | 6.63  (6.30, 6.96) | 7.12  (6.76, 7.47) | 6.75  (6.47, 7.03) | 6.59  (6.26, 6.96) | 6.61  (6.29, 6.93) | 6.63  (6.28, 6.97) | 6.57  (6.17, 6.94) | 0.12  (0.11, 0.14) |  |
|  | Overestimators | 5.81  (5.33, 6.29) | 6.68  (5.70, 7.70) | 6.04  (4.98, 6.94) | 5.86  (4.82, 6.96) | 7.26  (6.35, 8.32) | 6.86  (6.21, 7.44) | 5.93  (5.16, 6.62) | 0.18  (0.14, 0.22) |  |
| Sleep latency, min | Underestimators | 41.50  (14.50, 84.51) | 27.61  (15.07, 44.39) | 29.50  (14.42, 47.35) | 23.03  (12.80, 36.54) | 16.90  (9.67, 24.80) | 19.32  (11.82, 27.25) | 25.27  (14.19, 39.54) | 0.62  (0.49, 0.74) | 0.795 |
|  | Concordant estimators | 14.21  (11.01, 17.88) | 11.97  (9.15, 15.40) | 13.69  (10.36, 17.70) | 11.50  (8.93, 14.62) | 18.54  (13.58, 23.58) | 17.43  (12.60, 23.24) | 15.93  (10.79, 23.16) | 0.63  (0.56, 0.70) |  |
|  | Overestimators | 17.20  (11.65, 22.55) | 22.91  (12.36, 36.55) | 25.77  (11.27, 44.09) | 26.91  (14.04, 46.14) | 24.68  (15.73, 36.05) | 40.04  (12.54, 81.06) | 19.30  (11.65, 27.05) | 0.69  (0.51, 0.86) |  |
| Sleep efficiency, % | Underestimators | 78.89  (66.50, 88.96) | 87.44  (82.94, 91.39) | 86.65  (82.55, 90.33) | 85.90  (81.51, 89.99) | 85.84  (76.91, 91.79) | 87.89  (82.47, 92.30) | 83.70  (76.86, 89.73) | 0.11  (0.06, 0.17) | 0.024 |
|  | Concordant estimators | 89.39  (87.42, 91.20) | 89.20  (87.60, 90.84) | 88.94  (86.82, 90.93) | 89.52  (87.50, 91.24) | 88.86  (86.78, 90.77) | 87.16  (84.62, 89.64) | 89.96  (88.27, 91.85) | 0.06  (0.05, 0.07) |  |
|  | Overestimators | 89.02  (86.47, 91.82) | 90.38  (87.25, 92.83) | 91.58  (88.70, 94.57) | 85.24  (79.71, 90.83) | 83.89  (74.41, 90.88) | 83.68  (74.19, 90.73) | 88.81  (80.68, 94.40) | 0.10  (0.05, 0.15) |  |
| REM latency, min | Underestimators | 72.96  (57.71, 89.97) | 51.11  (34.32, 68.50) | 63.08  (48.61, 77.08) | 70.57  (60.76, 81.41) | 57.97  (41.93, 74.97) | 65.79  (50.71, 78.72) | 70.19  (53.96, 85.77) | 0.44  (0.29, 0.61) | 0.192 |
|  | Concordant estimators | 67.21  (59.48, 75.26) | 62.08  (52.58, 71.39) | 68.85  (60.75, 76.73) | 67.67  (60.05, 75.63) | 67.17  (59.22, 77.25) | 69.13  (61.58, 77.17) | 66.65  (56.77, 76.64) | 0.31  (0.26, 0.38) |  |
|  | Overestimators | 63.80  (56.50, 70.85) | 59.27  (51.50, 67.14) | 86.68  (64.63, 110.55) | 92.82  (71.18, 126.14) | 81.68  (57.77, 105.14) | 60.08  (47.41, 71.63) | 77.05  (52.40, 103.91) | 0.38  (0.30, 0.46) |  |
| Arousal index, index | Underestimators | 16.39  (11.28, 23.89) | 13.31  (10.63, 16.44) | 13.25  (11.00, 15.45) | 14.20  (11.08, 17.55) | 14.38  (11.51, 17.10) | 13.37  (11.09, 16.02) | 14.39  (11.92, 17.01) | 0.25  (0.18, 0.34) | 0.218 |
|  | Concordant estimators | 12.81  (11.27, 14.50) | 13.21  (11.67, 14.81) | 12.82  (11.50, 14.25) | 12.53  (10.93, 14.26) | 12.62  (11.10, 14.19) | 12.49  (11.07, 13.93) | 12.19  (10.72, 13.67) | 0.23  (0.20, 0.26) |  |
|  | Overestimators | 13.86  (10.05, 17.72) | 10.97  (7.69, 14.87) | 10.78  (7.24, 14.93) | 9.22  (6.86, 11.56) | 12.44  (9.23, 16.79) | 10.99  (8.54, 13.99) | 9.60  (7.24, 11.95) | 0.30  (0.25, 0.35) |  |
| N1, % | Underestimators | 9.00  (7.16, 10.81) | 8.99  (7.13, 11.13) | 9.67  (8.20, 11.24) | 10.07  (7.93, 12.34) | 9.26  (7.53, 11.35) | 8.64  (7.31, 10.21) | 8.61  (6.96, 10.43) | 0.32  (0.26, 0.39) | 0.340 |
|  | Concordant estimators | 8.18  (7.05, 9.42) | 8.50  (7.23, 9.86) | 8.36  (7.10, 9.77) | 8.18  (6.85, 9.52) | 8.02  (6.74, 9.29) | 7.81  (6.49, 9.27) | 8.06  (6.43, 9.77) | 0.35  (0.31, 0.39) |  |
|  | Overestimators | 11.33  (7.90, 14.92) | 7.61  (5.50, 9.98) | 7.91  (5.53, 10.54) | 7.18  (4.73, 9.32) | 9.15  (7.37, 11.17) | 8.32  (5.77, 10.52) | 7.14  (5.38, 8.88) | 0.41  (0.34, 0.48) |  |
| N2, % | Underestimators | 42.23  (35.28, 48.30) | 48.55  (44.99, 52.18) | 47.67  (44.10, 51.70) | 46.58  (42.45, 50.59) | 46.37  (40.61, 51.40) | 49.08  (46.25, 51.89) | 43.82  (39.17, 48.47) | 0.15  (0.10, 0.20) | 0.399 |
|  | Concordant estimators | 47.50  (45.53, 49.69) | 47.33  (44.61, 50.11) | 45.90  (43.39, 48.32) | 46.97  (44.82, 49.08) | 46.23  (43.92, 48.62) | 47.33  (44.97, 49.98) | 47.45  (45.63, 49.36) | 0.13  (0.11, 0.15) |  |
|  | Overestimators | 43.97  (40.77, 46.91) | 47.54  (43.04, 51.64) | 47.90  (43.05, 52.46) | 46.03  (40.65, 50.74) | 46.45  (39.58, 53.33) | 41.19  (33.01, 48.66) | 47.25  (42.75, 51.39) | 0.16  (0.11, 0.21) |  |
| N3, % | Underestimators | 6.06  (2.62, 9.99) | 5.76  (2.60, 9.34) | 8.48  (4.22, 12.75) | 7.89  (4.02, 12.13) | 8.61  (4.69, 12.98) | 8.17  (4.64, 11.53) | 9.41  (4.49, 14.33) | 0.69  (0.50, 0.97) | 0.547 |
|  | Concordant estimators | 10.91  (8.90, 13.00) | 10.22  (7.89, 12.82) | 10.15  (8.03, 13.33) | 11.03  (8.63, 13.33) | 10.64  (8.06, 13.32) | 9.77  (7.82, 11.79) | 10.07  (7.57, 13.08) | 0.73  (0.56, 0.91) |  |
|  | Overestimators | 9.79  (5.99, 14.03) | 11.41  (6.63, 16.19) | 9.94  (5.17, 15.75) | 12.87  (8.00, 17.89) | 10.79  (5.44, 16.34) | 9.09  (3.96, 14.64) | 11.15  (7.05, 15.93) | 0.52  (0.37, 0.68) |  |
| WASO, % | Underestimators | 21.45  (16.80, 25.35) | 24.83  (21.31, 28.08) | 19.93  (16.98, 23.06) | 22.77  (20.54, 25.08) | 21.47  (18.00, 24.56) | 21.75  (18.45, 25.23) | 20.94  (17.76, 24.13) | 0.24  (0.18, 0.29) | 0.908 |
|  | Concordant estimators | 22.88  (21.11, 24.60) | 23.34  (21.41, 25.09) | 24.26  (22.36, 26.27) | 23.61  (21.81, 25.26) | 23.11  (21.30, 24.68) | 22.91  (21.24, 24.59) | 23.76  (21.70, 25.72) | 0.22  (0.20, 0.26) |  |
|  | Overestimators | 23.85  (20.27, 27.48) | 23.92  (21.09, 27.22) | 21.53  (18.37, 24.41) | 21.80  (18.71, 24.19) | 18.64  (15.44, 22.43) | 21.95  (19.23, 24.45) | 25.65  (21.60, 29.86) | 0.23  (0.19, 0.28) |  |
| Total delta power during N1, μV^2^ | Underestimators | 85.11  (52.12, 129.03) | 53.06  (42.29, 64.73) | 60.47  (44.30, 76.68) | 74.83  (45.46, 118.27) | 59.79  (43.54, 77.79) | 70.12  (51.06, 95.05) | 57.78  (49.00, 67.05) | 0.29  (0.24, 0.34) | 0.918 |
|  | Concordant estimators | 73.55  (63.80, 84.80) | 67.60  (57.94, 78.30) | 70.53  (59.95, 82.12) | 63.83  (54.82, 73.70) | 69.81  (60.01, 80.21) | 63.98  (53.19, 77.89) | 64.77  (55.75, 74.78) | 0.28  (0.25, 0.30) |  |
|  | Overestimators | 61.21  (48.16, 76.02) | 57.09  (41.21, 75.67) | 60.16  (46.46, 76.99) | 51.13  (42.64, 76.99) | 60.20  (45.35, 80.95) | 54.78  (44.94, 65.15) | 46.31  (37.92, 55.15) | 0.28  (0.23, 0.34) |  |
| Total delta power during N2, μV^2^ | Underestimators | 112.77  (83.45, 146.81) | 85.06  (67.98, 103.12) | 99.46  (76.80, 127.64) | 113.81  (79.04, 158.07) | 100.84  (81.56, 125.32) | 108.41  (82.93, 138.04) | 101.92  (88.42, 116.95) | 0.24  (0.18, 0.31) | 0.202 |
|  | Concordant estimators | 115.47  (104.08, 126.60) | 114.40  (102.17, 129.01) | 113.12  (98.41, 129.03) | 108.02  (96.57, 118.34) | 113.02  (101.69, 124.46) | 106.98  (93.73, 122.73) | 113.63  (97.31, 131.47) | 0.21  (0.18, 0.23) |  |
|  | Overestimators | 96.07  (76.13, 118.50) | 78.74  (63.20, 97.33) | 103.89  (78.21, 142.34) | 91.23  (76.28, 114.29) | 100.24  (78.11, 130.99) | 92.38  (76.04, 110.91) | 74.70  (63.04, 86.84) | 0.17  (0.13, 0.21) |  |
| Total delta power during N3, μV^2^ | Underestimators | 535.76  (357.96, 780.82) | 450.61  (348.84, 544.43) | 545.80  (413.26, 703.78) | 684.25  (377.35, 1083.9) | 604.54  (413.97, 921.09) | 567.75  (419.01, 756.56) | 586.12  (410.61, 848.84) | 0.37  (0.24, 0.51) | 0.003 |
|  | Concordant estimators | 436.62  (386.81, 488.22) | 481.79  (387.28, 593.00) | 472.59  (402.44, 556.01) | 468.95  (386.30, 587.38) | 461.86  (371.09, 582.83) | 423.26  (374.97, 476.12) | 515.07  (400.37, 681.11) | 0.18  (0.14, 0.22) |  |
|  | Overestimators | 457.58  (305.71, 629.41) | 373.27  (269.79, 454.40) | 452.58  (317.45, 627.34) | 449.16  (337.12, 588.88) | 472.37  (341.17, 621.10) | 479.52  (356.99, 606.09) | 388.92  (299.49, 494.20) | 0.23  (0.17, 0.31) |  |
| Total delta power during REM, μV^2^ | Underestimators | 47.83  (40.34, 56.25) | 42.34  (34.36, 52.25) | 41.22  (35.09, 47.73) | 43.10  (33.02, 55.86) | 41.74  (36.20, 47.87) | 43.48  (36.03, 51.34) | 44.38  (35.48, 53.89) | 0.23  (0.19, 0.27) | 0.333 |
|  | Concordant estimators | 51.19  (45.85, 56.40) | 48.04  (43.22, 53.44) | 44.46  (38.48, 50.51) | 41.02 (36.78, 45.86) | 45.52  (40.79, 50.86) | 42.85  (37.99, 47.85) | 40.50  (36.33, 44.88) | 0.25  (0.22, 0.28) |  |
|  | Overestimators | 51.77  (39.23, 66.60) | 43.95  (31.03, 60.83) | 52.79  (33.55, 85.39) | 45.56  (30.97, 67.24) | 49.80  (36.46, 65.20) | 45.75  (33.13, 63.65) | 38.55  (31.31, 46.45) | 0.20  (0.13, 0.27) |  |

Note: Data are presented as mean (95% confidence interval) for each night and coefficient of variation (CV) across seven nights. P-values are derived from one-way ANOVA comparing CVs across groups. Groups were classified based on baseline Sleep Perception Index (SPI): underestimators, concordant estimators, and overestimators. TST, total sleep time; WASO, wake after sleep onset; REM, rapid eye movement sleep; CV, coefficient of variation; CI, confidence interval; SPI, Sleep Perception Index.
